# HSP90 is part of a protein complex with the L polymerase of Rift Valley fever phlebovirus and prevents its degradation by the proteasome during the viral genome replication/transcription stage

**DOI:** 10.3389/fcimb.2024.1331755

**Published:** 2024-05-10

**Authors:** Farhang Alem, Ashwini Brahms, Kaori Tarasaki, Samson Omole, Kylene Kehn-Hall, Connie S. Schmaljohn, Sina Bavari, Shinji Makino, Ramin M. Hakami

**Affiliations:** ^1^ School of Systems Biology, George Mason University, Manassas, VA, United States; ^2^ Department of Microbiology and Immunology, The University of Texas Medical Branch, Galveston, TX, United States; ^3^ Integrated Research Facility at Fort Detrick, Division of Clinical Research, National Institute of Allergy and Infectious Diseases, National Institutes of Health, Fort Detrick, Frederick, MD, United States; ^4^ Tonix Pharmaceuticals, Frederick, MD, United States; ^5^ Center for Infectious Disease Research, George Mason University, Manassas, VA, United States

**Keywords:** Rift Valley fever virus, RNA-dependent RNA polymerase, L protein, Hsp90, heat shock protein, protein stability, proteasome, protein degradation

## Abstract

The mosquito-borne Rift Valley fever virus (RVFV) from the *Phenuiviridae* family is a single-stranded RNA virus that causes the re-emerging zoonotic disease Rift Valley fever (RVF). Classified as a Category A agent by the NIH, RVFV infection can cause debilitating disease or death in humans and lead to devastating economic impacts by causing abortion storms in pregnant cattle. In a previous study, we showed that the host chaperone protein HSP90 is an RVFV-associated host factor that plays a critical role post viral entry, during the active phase of viral genome replication/transcription. In this study, we have elucidated the molecular mechanisms behind the regulatory effect of HSP90 during infection with RVFV. Our results demonstrate that during the early infection phase, host HSP90 associates with the viral RNA-dependent RNA polymerase (L protein) and prevents its degradation through the proteasome, resulting in increased viral replication.

## Introduction

Rift Valley fever virus (RVFV) is a member of the *Phenuiviridae* family that is primarily transmitted by mosquitos and has caused disease outbreaks in Africa and the Arabian Peninsula (Mroz et al., 2017; Samy et al., 2017; Sang et al., 2017). Rift Valley fever (RVF) is a re-emerging zoonotic disease that mainly affects ruminants but can also be transmissible to people (Mroz et al., 2017). Due to its virulence, devastating economic impact, and ease of aerosolization, the virus is considered a potential biothreat and is listed as a select agent by both the HHS and USDA, and has been classified by NIH/NIAID as an agent of highest concern (Category A agent). Outbreaks of the virus among cattle can have a devastating economical effect, causing abortion storms in pregnant animals (Bird and McElroy, 2016; Linthicum et al., 2016; Nanyingi et al., 2017). An outbreak in Somalia in 1997 caused the collapse of their livestock industry, and another outbreak in Kenya in 2006/2007 resulted in145,000 human cases of RVF (Pourrut et al., 2010; Dar et al., 2013; Himeidan et al., 2014). Transmission from animals to people is primarily through mosquito vectors, but can also occur through direct contact with body fluids of infected animals (Samy et al., 2017). Human infections in endemic regions are either asymptomatic or mainly cause mild illness in infected individuals; however, a small percentage of infected individuals develop more severe and life-threatening symptoms that range from optical lesions to encephalitis and hemorrhagic fever (Mroz et al., 2017). Furthermore, emergence of the disease in non-endemic areas can result in high rates of human death. For instance, the RVFV outbreak of 2000 in Egypt resulted in a 14% case fatality rate (Himeidan et al., 2014). Currently, there are no available vaccines or therapeutics against RVF, and given the threats it poses, further research into effective vaccine and therapeutic developments are urgently needed.

RVFV is an enveloped single-stranded RNA virus with a tripartite genome consisting of S (small), M (medium), and L (large) segments. The S segment encodes the structural nucleoprotein N and a non-structural protein NSs. The M segment encodes for the glycoproteins Gn and Gc, as well as another non-structural protein called NSm and 78 kDa protein. Lastly, the L segment encodes for the RNA-dependent RNA polymerase L protein (Ikegami and Makino, 2011). In general, viruses have a relatively simple proteome and as such require the help of host cell machinery to carry out most of their functions such as replication and release from the cell. For example, unrelated to the HSP90 role, RVFV NSs protein has been shown to cause increased phosphorylation of p53, leading to decreased cell death and increased viral production (Austin et al., 2012). Another example is our demonstration that host PP1 plays a role in early life cycle of RVFV replication (Baer et al., 2016).

HSP90 has also been shown to be an important host factor that influences the replication of a number of viruses. Heat Shock Protein 90 (HSP90) is a highly conserved ubiquitous ATP-dependent molecular chaperone that is crucial for proper folding and maturation of over 200 identified client proteins (Wandinger et al., 2008), which range from steroid receptors to transcription factors, protein kinases, and oncogenes (Pratt and Toft, 2003). HSP90 forms a complex with HSP70 and other chaperone proteins in order to stabilize and activate client proteins. Studies have shown that about 60% of all kinases and 7% of transcription factors associate with HSP90. The properties of HSP90 have been shown to influence a variety of viral infections. For example, during HIV infection, HSP90 can reactivate a latent infection by maintaining the function of the positive Transcription Elongation Factor b (Pan et al., 2016), and in Vaccinia virus the inhibition of HSP90 by the viral protein 4a blocks viral replication (Hung et al., 2002). Furthermore, for several viruses that similar to RVFV are non-retroviral single stranded RNA (ssRNA) viruses, it has been demonstrated that HSP90 is able to control viral polymerase function. For example, for the influenza virus, HSP90 has been shown to interact with the polymerase protein PB2 subunit (Jirakanwisal et al., 2015), and in flock house virus, HSP90 can affect the stability and localization of the viral RNA polymerase (Kampmueller and Miller, 2005; Castorena et al., 2007). HSP90 is also required by the polio virus for both proper protein folding of the viral capsid protein and proper viral polymerase functions (Geller et al., 2007). In another study, HSP90 was shown to associate with the viral L polymerase of the Mumps virus to aid in transcription and replication (Katoh et al., 2017). Several additional examples exist as well. Calu-3 cells treated with 17-AAG and other HSP90 inhibitors showed a 50-70% reduction in viral RNA levels 16 hours post infection with SARS-CoV-2 (Lubkowska et al., 2021). Also, for respiratory syncytial virus (RSV) infection, inhibition of HSP90 led to the degradation of viral proteins, including the RdRp L protein that is vital for viral replication (Geller et al., 2007). Additionally, antibodies against HSP90 and HSP70 were shown to prevent dengue virus (DENV) infection in neuroblastoma cells (Geller et al., 2007). Given these various findings, HSP90 has gained increased attention for its regulatory role during viral functions and its role in host stress response (Lubkowska et al., 2021). In addition, inhibitors of HSP90 have been used to treat a host of other diseases. A search of clinicaltrials.gov returned 102 clinical trials using HSP90 inhibitors to treat or prevent diseases such as psoriasis, diabetic atherosclerosis, hidradenitis suppurativa, and various types of cancer.

In a previous study, we demonstrated that HSP90 is a RVFV-associated host factor that also plays an important role during the viral genome replication/transcription phase of the life cycle of this virus (Nuss et al., 2014). We showed that both the siRNA knockdown of HSP90AB1 and chemical inhibition of HSP90 using specific inhibitors results in significant lowering of viral levels, without influencing the functional integrity of the virions that are nevertheless produced and released from the cell. Furthermore, through time-of-addition inhibitor studies, we demonstrated that the HSP90 effect is post viral entry and is most dramatic between 2 hours post infection (hpi) and 4 hpi, with the effect being noticeably less at 8 hpi but still statistically significant (it became statistically insignificant by 14 hpi). This time period of most significant effect (2-4 hpi) corresponds to the known active phase of RVFV genome replication/transcription, since following RVFV infection of cells, viral genome replication and transcription is already under way by 6 hpi and the newly assembled virions begin to egress from the cell starting at 10 hpi. In this study, we have performed mechanistic studies to elucidate how HSP90 is involved in the regulation of RVFV genome transcription and replication. Based on the timing of HSP90 effects during RVFV infection and previous studies reporting on HSP90 interaction with the polymerase of other ssRNA viruses, we hypothesized that a similar mechanism of action exists for RVFV infection. Our results reported here demonstrate that HSP90 function is indeed critical for the stability of the viral L polymerase protein and thus for the ability of viral replication to proceed.

## Materials and methods

### Virus infection

Cells were grown at 37°C in 5% CO_2_.Vero cells (ATCC) were grown and maintained in Dulbecco’s Modified Minimum Essential Medium (DMEM) supplemented with 10% heat-inactivated Fetal Bovine Serum (FBS), 1% penicillin/streptomycin, and 1% L-glutamine. Human liver hepatocellular carcinoma cell line (HepG2) was maintained in Eagle’s Minimum Essential Medium (EMEM; Lonza) supplemented with 10% heat-inactivated fetal bovine serum (FBS; Life Technologies) and 100 U/ml penicillin-streptomycin (Life Technologies), or in a 1:1 ratio mixture of DMEM and Ham’s F12 medium supplemented with 10% heat-inactivated FBS, 1% penicillin/streptomycin, and 1% L-glutamine. For virus infection Vero cells were used as previously described (Nuss et al., 2014; Alem et al., 2021) for production of either MP12 strain (Nuss et al., 2014) or MP12-L-V5, NSs-FLAG strain (Alem et al., 2021), which is a tagged version of MP12 that encodes FLAG-tagged NSs (NSs-FLAG) and V5-tagged L protein (L-V5). Cultured Vero cells or HepG2 cells were infected with either regular MP12, or with tagged MP12, at multiplicity of infection (MOI) of 3, by overlaying the cells with a suspension of virus (2.5 x 10^5^ cells per well for a 6-well plate; or, 10^5^ cells per well for a 12-well plate; or, 10^4^ cells per well in a 96-well plate) followed by incubation for 1 hour at 37°C, 5% CO_2_. Subsequently, the inoculum was removed and after washing the cells twice with Phosphate Buffer Saline (PBS) they were incubated with supplemented culture media until the specified collection time points.

For infection studies with ZH501 in the presence of HSP90 inhibitors, HepG2 cells were seeded at a density of 7.5 x10^3^ cells per well in black, clear-bottom 96-well plates (Greiner Bio-One), incubated at 37°C with 5% CO_2_, and then used for experiments one day later. RVFV strain ZH501 was propagated in Vero cell monolayers and infected cell culture supernatant was clarified by centrifugation, divided into aliquots, and stored at -80°C prior to use. All infections utilizing RVFV ZH501 were performed in a biosafety level 3 (BSL-3) laboratory.

### Heat shock assay

Vero cells were seeded into two 96-well plates at a cell density of 10^4^ cells per well and incubated overnight at 37°C and 5% CO_2_. The following day, one of the 96 well plates was heat shocked at 48°C for 1 hr while the second plate was left at 37°C. Following the heat shock treatment, cells were either treated with 17-AAG (1 μM final concentration) for 1 hour, or treated with an equal volume of DMSO vehicle as control, and were subsequently infected with RVFV MP12-L-V5, NSs-FLAG strain (MOI 3). After infection, 17-AAG-supplemented complete media was added back to the wells, and both plates were then incubated overnight. The following day, the culture supernatants from both plates were collected at 24 hpi and quantified by plaque assay for determination of plaque forming unit counts (PFU/ml) as described below. Three biological replicates were performed.

### Plaque assay

Vero cells were seeded in a 12-well plate at a density of 10^6^ cells per well and incubated for 24 hrs at 37°C and 5% CO_2_. Supernatants from the heat shock study described above were serially diluted by making 10-fold dilutions in DMEM (10^-3^ to 10^-8^). Subsequently, 200 μl of each dilution was used to infect each well of the 12-well plate in duplicate. The infection was carried out for 1 hour, followed by the addition of a 3 ml overlay of a 1:1 mixture of 0.6% agarose in diH_2_O and 2X EMEM supplemented with 5% FBS, 2% penicillin/streptomycin, 1% L-Glutamine, 1% Sodium Pyruvate, and 1% non-essential amino acids (NEAA). The plates were incubated at 37°C, 5% CO_2_ for 72 hrs, and the cells were subsequently fixed using 10% formaldehyde. Agarose plugs were removed, and cells were stained with 1% crystal violet dye in 20% methanol in diH_2_O. The average number of plaques was enumerated for each treatment, and the following equation was used to quantify viral titer: PFU/ml = Average number of plaques multiplied by dilution factor of 5.

### Western blot analysis

At desired time points following infection, the growth media was removed and after washing the cells with PBS they were lysed with Blue Lysis Buffer and boiled for 10 minutes. The Blue Lysis Buffer consists of 1:1 mixture of T-PER reagent (Pierce, IL) and 2X Tris-glycine SDS sample buffer (Novex, Invitrogen), containing 33 mM DTT and phosphatase and protease inhibitor cocktails (1X Halt cocktail, Pierce). Per sample, 25 µL or 30 µL of the whole cell lysate in Blue Lysis Buffer was separated on NuPAGE 4-12% Bis-Tris gels (Invitrogen) and the proteins were then transferred to membrane by either overnight wet transfer at 70 mA, or a 2-hour wet transfer at 250 mA at 4°C, using an XCell II Blot Module (Invitrogen). After the transfer, the membrane was blocked using 3% dehydrated milk solution in 1X PBS containing 0.1% Tween-20 (PBS-T) for at least an hour at room temperature. The primary antibodies diluted in PBS-T at 1:1000 were then added to the blot and incubated overnight (the β-Actin primary antibody was diluted 1:10,000 and the blot was incubated with it for 30 minutes at room temperature). The membrane was then washed 4 times with PBS-T (5 minutes per wash) and subsequently incubated with secondary HRP-coupled goat anti-mouse antibody (1:1000 dilution in PBS-T) for 2 hours. The membrane was then washed 4 times with PBS-T (5 minutes per wash), and the western blot was imaged using SuperSignal West Femto Maximum Sensitivity Substrate kit (Thermo Fisher Scientific) in a Bio-Rad Molecular Imager ChemiDoc XRS System (Bio-Rad). The primary antibodies used were: anti-RVFV N Protein (a gracious gift from Dr. Connie Schmaljohn, USAMRIID); HRP-conjugated anti-β-actin (Cat# ab49900-100, Abcam); anti-FLAG (Cat# F1804, Sigma); anti-V5 (Cat# MCA1360, Serotec); anti-PKR D-20 (Cat# sc-708, Santa Cruz); and, anti-HSP90 (Cat#4877, Cell Signaling).

### RT-qPCR

Cultured cells were infected with RVFV MP12 (MOI 0.1) for 1 hr as described above. At the specified time point, intracellular RNA extraction was performed using the RNEasy kit (QIAGEN) per manufacturer’s instructions. Extracted RNA was converted to cDNA using the High Capacity RNA-to-cDNA kit (Invitrogen) according to manufacturer’s protocol. For q-PCR, the template cDNA was added to a 20 µL reaction with SYBR^®^ GREEN PCR master mix (Invitrogen) and 0.2 µM primer. cDNA was amplified using the ABI Prism 7000 (1 cycle -95°C for 10min, 40 cycles- 95°C for 15sec, and 60°C for 1min). Fold changes were calculated relative to β-actin using the ΔΔCt method. Primer sequences: [RVFV L Polymerase Forward: GGT GGC ATG TTC AAT CCT TT; Tm = 53.6°C] [RVFV L Polymerase Reverse: GCA TTC TGG GAA GTT CTG GA; Tm = 54.8°C] [RVFV NSs Forward: TCT GAA AGA AGC CAT ATC CT; Tm = 50.6°C] [RVFV NSs Reverse: CTC GCT ATC ATC CTG TGT AA; Tm = 51.6°C] [RVFV N Forward: CAT GGT GGA TCC TTC TCT AC; Tm = 52°C] [RVFV N Reverse: CTA TTC ACT GCT GCA TTC AT; Tm = 50.5°C] [RVFV Gn Forward: AAA GGA ACA ATG GAC TCT GGT CA; Tm = 56.3°C] [RVFV Gn Reverse: CAC TTC TTA CTA CCA TGT CCT CCA AT; Tm = 56.2°C]. Three biological replicates were performed.

### Cell viability assay

At the specified time point, cell viability assay was performed for treated cells using CellTiter-Glo Cell Luminescent Viability Assay (Promega), according to the manufacturer’s instructions. Briefly, 10^4^ cells in 100 μl culture media were plated into opaque-walled 96 well plates overnight to allow them to adhere. Cells were then treated either with 17-AAG or radicicol inhibitors at the various concentrations indicated in the manuscript, or DMSO as control, and incubated overnight in a cell culture incubator (37°C, 5% CO_2_). The following day, the culture plates were removed from the incubator and were left at room temperature and allowed to adapt. Subsequently, to each well, a volume of CellTiter-Glo reagent equivalent to the volume of the media in the well was added. The plate was then placed on an orbital plate shaker for 2 minutes and subsequently incubated at room temperature for 10 minutes. Viability was assessed by luminescence detection using the DTX 880 multimode detector (Beckman Coulter). Three biological replicates were performed.

### Co-immunoprecipitation studies

Vero cells were cultured with or without 17-AAG inhibition (1 μM final concentration), and subsequently infected with RVFV MP12-L-V5, NSs-FLAG (MOI 3) as previously described. At 24 hpi, cells were washed and lysed using a clear lysis buffer (50mM Tris-HCl pH 7.4, 120mM NaCl, 5mM EDTA, 0.5% NP-40, 50mM NaF, 0.2mM Na_3_VO_4_ and 1X Protease Inhibitor Cocktail) to prevent disruption of protein-protein interactions. Cell lysates (1mg total protein) were incubated with magnetic Dynabeads derivatized with the antibody of interest (either anti-HSP90 or anti-V5) for 24 hours, gently rocking at 4°C. Subsequently, the tubes were placed into magnetic holders to isolate the beads bound with the antibody-antigen complexes and the supernatants were discarded. The beads were washed three times with PBS, and the samples were then eluted off the beads using clear lysis buffer. The eluates were subjected to western blot analysis using either α-HSP90, α-V5, or α-FLAG antibodies. Three biological replicates were performed.

### Immunofluorescence staining

Vero cells were seeded into an 8 chamber slide at a concentration of 1 x 10^3^ cells per well and grown overnight. Cells were infected with RVFV MP12-L-V5, NSs-FLAG (MOI 3) for 24 hours and then fixed with 4% paraformaldehyde. The cells were subsequently permeabilized with 0.5% Triton X-100 in PBS for 20 minutes and then washed 2x with PBS and blocked in 3% BSA in PBS for 30 minutes at room temp. Cells were subsequently incubated with primary antibody (α-HSP90 and α-V5 antibodies) in fresh blocking buffer for 1 hr and then washed three times (3 minutes per wash) with 1X PBS containing 0.1% Triton X-100 (Sigma-Aldrich). Using the same treatment procedure as the primary antibody, the cells were then treated with diluted fluorescently-labeled secondary antibody and excess secondary antibody was washed off. The cells were then mounted on a glass microscope slide using Fluoromount-G mounting medium with DAPI (VWR) with cover slip placed on top. Fluorescence microscopy was carried out to visualize the cells using a Nikon AX R Confocal microscope. Three biological replicates were performed.

### Pulse-chase studies

Vero cells were treated with DMSO or 1 µM of 17-AAG (LC Laboratories) 1 hr prior to infection. DMSO or 17-AAG were added in all inoculum and medium used in following procedures. The cells were infected with the L-V5, NSs-FLAG virus (MOI 3). At 3 hpi, the culture medium was replaced with methionine/cysteine–free medium. After starvation for 30 minutes, cells were labeled with 200 μCi/ml (for immunoprecipitation analysis using anti-MP12 antibody) or 400 μCi/ml (for immunoprecipitation analysis of NSs) of Tran^35^S-label (1,000 Ci/mmol; MP Biomedicals) for 30 minutes. After pulse labeling, the cells were incubated with complete medium supplemented with methionine/cysteine for various periods of time in the absence or presence of 17-AAG. The cells were lysed by using cell lysis buffer (1% TritonX-100, 0.5% Na-deoxycholate, 0.1% SDS and protease inhibitor in PBS). Immunoprecipitation (IP) was performed by using Dynabeads protein G (Thermo Fisher Scientific) with anti-MP12 mouse serum (kindly provided by Dr. R.B. Tesh, UTMB) for Gn/Gc and N proteins, or anti-FLAG tag mouse monoclonal antibody (Cell Signaling Technology) for FLAG-tagged NSs protein, according to the manufacturer’s protocol. V5-tagged L protein was immunoprecipitated by using anti-V5-tag mAb-Magnetic Beads (Medical and Biological Laboratories) according to the manufacturer’s protocol. Samples were analyzed by autoradiography. For densitometry analysis, films were exposed with the samples for various times. The band intensities of viral proteins were measured by ImageJ software. Four biological replicates of pulse chase and IP were performed using anti-MP12 antibody and two biological replicates were performed using the anti-V5 and anti-FLAG antibodies.

### HSP90 inhibitor studies

The HSP90 inhibitor 17-AAG was obtained from LC Laboratories or Tocris Biosciences. Radicicol was obtained from Tocris Biosciences, and dimethyl sulfoxide (DMSO) was purchased from Sigma-Aldrich. Stock solutions of 1 mM and 10 mM concentration were prepared by dissolving the inhibitors in DMSO. The stocks were divided into aliquots and stored at -20°C prior to use. For studies with the MP12 strain, dilutions were prepared in supplemented culture medium to achieve a final concentration of 1 μM 17-AAG for pre-treating cells with the inhibitor for 1 hr. Subsequently, the inhibitor-containing medium was removed and the cells were either mock infected, or infected with either MP12 strain (for RT-qPCR studies) or MP12-L-V5, NSs-FLAG (for western blot analysis) for 1 hr as described above. Following removal of the inoculum and washing of the cells with PBS, fresh medium was added back to the cells (either with or without inhibitor), and samples were collected at the 4 hrs, 6 hrs, 8 hrs, 10 hrs, and 24 hrs post-infection time points. Intracellular RNA was extracted and converted to cDNA for RT-qPCR analysis as described above in order to quantify viral RNA levels. In addition, whole cell lysates were analyzed by western blot analysis of viral proteins as described above. Three biological replicates were performed. For studies with ZH501, dilution series of each compound were prepared in EMEM supplemented with 10% FBS and 100 U/ml penicillin-streptomycin. The existing media was removed from cell culture plates and replaced with medium containing the inhibitor either at 2 hrs before infection, or at 4 hrs or 8 hrs post infection, and maintained for the duration of the experiment. To infect the cells (MOI 5), the existing cell culture medium was removed from the wells and replaced with virus-containing culture medium. For cultures pretreated with compounds prior to infection, the respective inhibitor was also added to the virus inoculum. Infections were allowed to proceed for 1 hr at 37°C, and then the inoculum was removed and fresh medium was added (either with or without inhibitor). Three biological replicates were performed.

### Measurement of RVFV infection level by high content imaging

At approximately 20 hrs post infection with RVFV ZH501, the medium was removed from HepG2 cell cultures and the plates were submerged in 10% buffered formalin for at least 24 hrs to fix the cell monolayers and to inactivate RVFV prior to removal from BSL-3. The plates were rinsed with PBS and then the cell monolayers were permeabilized with 0.1% Triton X-100 (Sigma-Aldrich) in PBS for 15 minutes. After further rinsing with PBS, the monolayers were blocked with 3% bovine serum albumin (BSA; Sigma-Aldrich) for 1 hr. Anti-RVFV N monoclonal antibody R3-1D8-1-1 was diluted in blocking buffer 1:1000 and then added to the cell monolayers for 1 hr. Unbound primary antibody was rinsed away with PBS. Anti-mouse Alexa Fluor 488 secondary antibody (Thermo Fisher Scientific) was diluted in blocking buffer 1:2000 and added to the cell monolayers for 1 hr in the dark. Unbound secondary antibody was rinsed away with PBS, and the cells were subsequently counterstained with Hoechst 33342 and HCS Cell Mask Red (Thermo Fisher Scientific). High-content quantitative imaging data were acquired and analyzed on an Opera confocal reader (model 3842-Quadruple Excitation High Sensitivity (QEHS), Perkin Elmer), at two exposures using a 20x air objective. Analysis of the images was performed using Columbus software (Perkin Elmer).

## Results

### Inhibition of HSP90 by 17-AAG causes a decrease in viral protein and RNA levels

**Figure 1:**
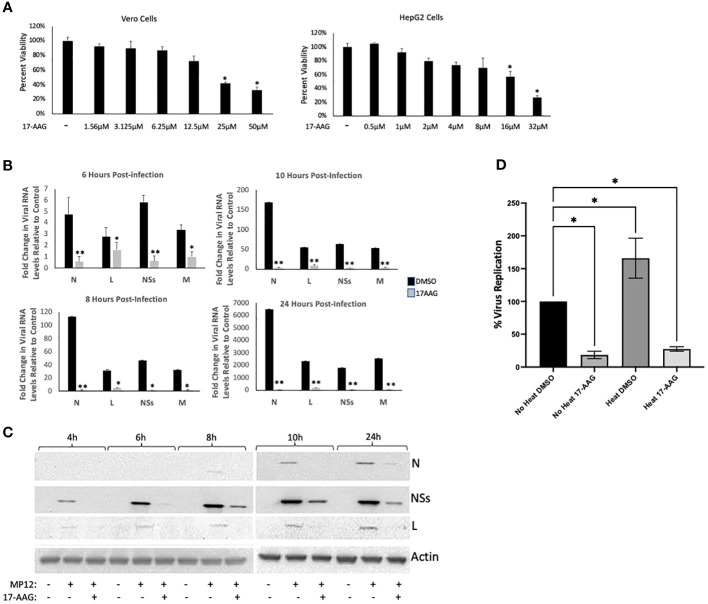
Analysis of 17-AAG Effects on Viral RNA and Proteins Levels over Time. **(A)** Vero cells (left panel) were treated with DMSO or 17-AAG at various concentrations (1.56 µM, 3.12 µM, 6.25 µM, 12.5 µM, 25 µM or 50 µM), and cell viability was measured at 24 hours post treatment (hpt). HepG2 (right panel) cells were also treated with DMSO or 17-AAG at the same various concentrations and cell viability was measured at 24 hours post treatment (hpt); *P ≤ 0.05 **(B)** Vero cells were treated with 17-AAG both pre- and post-infection with MP12 (MOI 3). Samples were collected at 4 hpi (data not shown), 6 hpi, 8 hpi, 10 hpi, and 24 hpi and intracellular RNA was extracted. RT-qPCR was then performed on the samples to determine the number of genomic copies of viral N, L, NSs and Gn relative to cellular β-Actin. Fold changes are relative to the DMSO-treated samples at 4 hours post infection. Mean values ±SEM from three biological replicates are shown. Statistical analysis was performed using Student’s t-test, comparing 17-AAG treatment with the DMSO control; *P ≤ 0.05; **P ≤ 0.01. **(C)** Vero cells were treated with either 1 µM 17-AAG, or with vehicle only (DMSO), both pre- and post-infection with MP12-L-V5, NSs-FLAG (MOI 3). Whole cell lysates were collected at 4 hpi, 6 hpi, 8 hpi, 10 hpi, and 24 hpi, and analyzed by western blot to determine the level of viral proteins. Anti-V5 antibody was used for detection of the L protein and anti-FLAG antibody was used for detection of NSs. N protein levels were assessed directly using anti-N antibody. **(D)** Vero cells were either heat shocked at 48˚C for 1 hour or left remaining at 37˚C. Cells were then treated with either 17-AAG, or with vehicle only (DMSO), both pre-and post-infection with MP12-L-V5, NSs-FLAG. Culture supernatants were collected at 24 hpi and their viral loads quantified by plaque assay; *P ≤ 0.05.

For these studies, we used the MP12 strain of RVFV, a live attenuated strain that has served as a well-established and informative model system for studying RVFV infection mechanisms (Austin et al., 2012; Nuss et al., 2014; Baer et al., 2016; Alem et al., 2021). Given our previous results that HSP90 is important during the active phase of RVFV genome replication/transcription, we hypothesized that HSP90 inhibition should result in significant reduction of viral RNA and protein levels. Accordingly, using the well-characterized and specific HSP90 inhibitor, 17-Allylamino-17-Demethoxygeldanamycin (17-AAG), we quantified the effect of HSP90 inhibition on both viral RNA and protein levels at various time points post infection (6, 8, 10, and 24 hpi). We initially tested the cytotoxicity of increasing concentrations of 17-AAG on Vero cells as well as HepG2 cells and found that at 1 μM 17-AAG has no cytotoxic effects on either cell line (**Figure 1A**). Therefore, all subsequent studies were done using a 1 μM concentration of 17-AAG. To assess viral genome replication, Vero cells were treated with 17-AAG both pre and post-infection. Beginning at 4 hpi and up to 24 hpi, total RNA was isolated from the cells and viral RNA levels for N, NSs, L, and Gn were quantified by RT-qPCR. Viral RNA levels for all four genes showed significant decreases in 17-AAG-treated cells as compared to the DMSO-treated control cells, starting at 6 hpi and continuing up to 24 hpi (**Figure 1B**). Viral protein levels were also assessed by western blot analysis (**Figure 1C**). The earliest time point at which the N protein was detectable was at 10 hpi. At this time point, 17-AAG treatment showed decreased levels of N protein compared to the DMSO-treated control and the trend continued through the last time point of the analysis (24 hpi). The levels of the NSs protein and the L polymerase were also significantly decreased by 17-AAG treatment compared to untreated controls, starting at the earliest time point analyzed (4 hpi) and continuing through the last time point of the analysis (24 hpi).

We also performed a heat shock study to provide a direct correlation to this set of data suggesting the importance of HSP90 chaperone activity for viral production. In this experiment, chaperone activity was induced by stressing the cells through exposure to a higher temperature (48°C for 1 hr), and subsequently the cells were either treated with 17-AAG or with DMSO vehicle before infection with RVFV. The following day, the viral loads were quantified by plaque assay to compare the heat shock conditions with the control conditions (no heat induction). The results show that heat induction of cells, which results in increased HSP90 chaperone activity, leads to generation of significantly higher viral loads (**Figure 1D**), providing evidence for the involvement of HSP90 chaperone activity. Together, these results establish that inhibition of HSP90 by 17-AAG causes significant decrease in both viral RNA and protein levels in infected Vero cells, and provides further evidence of a possible role for HSP90 in viral genome transcription and/or replication.

### HSP90 confers stability to the viral L polymerase

**Figure 2:**
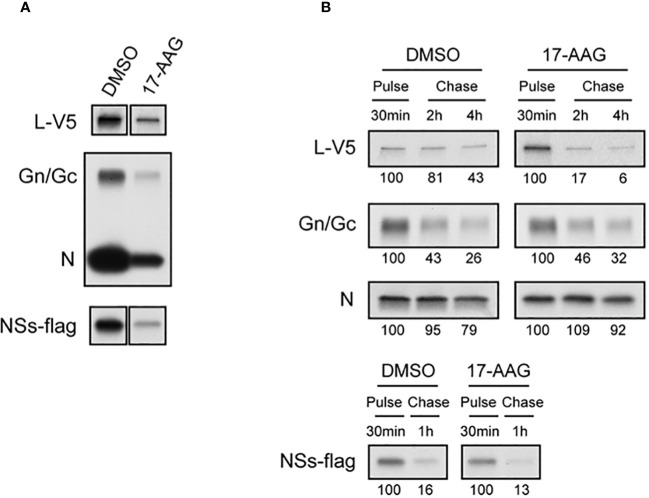
Effects of 17-AAG on the Synthesis and Stability of Viral Proteins. **(A)** Effect of 17-AAG on virus protein synthesis was analyzed. Vero cells were treated with DMSO or 1 µM of 17-AAG for 1 hr and infected with the MP12-L-V5, NSs-FLAG virus (MOI 3). DMSO or 17-AAG had been added in inoculum and medium until cells were harvested. The infected cells were pulsed with Tran^35^S-label for 30 minutes and harvested at 4 hpi. Viral proteins were immunoprecipitated by anti-V5 for L-V5 protein, anti-MP12 for Gn/Gc and N proteins, and anti-FLAG tag antibody for FLAG-tagged NSs protein, and analyzed by autoradiography. **(B)** Effects of 17-AAG on stability of viral proteins was analyzed. After pulse radiolabeling of the cells as described in **(A)**, cells were incubated in complete medium supplemented with methionine/cysteine for various periods of time indicated in the figure in the absence or presence of 17-AAG. Viral proteins were immunoprecipitated as described in **(A)**. The numbers below the panel represent signal intensities of each viral protein after pulse labeling and chase; signal of each protein after pulse labeling was set to 100. Representative data from independent experiments are shown.

The chaperone functions of HSP90 in protecting client proteins from degradation are well documented (Schopf et al., 2017). Since our results show that HSP90 inhibition causes a decrease in viral proteins and that heat induction of HSP90 chaperone activity significantly increases viral levels, we analyzed the effects of HSP90 on viral protein synthesis and stability by radiolabeling and pulse-chase analysis (**Figure 2**). Vero cells were treated with DMSO (control) or with 17-AAG, followed by infection with RFVF MP12-L-V5, NSs-FLAG and pulsed with Tran^35^S-label. Cells were harvested at 4 hpi and immunoprecipitated with either anti-V5, or anti-MP12, or anti-FLAG antibodies for pull down of the L protein, the Gn/Gc and N proteins, and the NSs protein respectively, followed by autoradiograph analysis (**Figure 2A**). As demonstrated by **Figure 2A**, effective radiolabeling and detection of viral proteins was achieved in Vero cells treated with either DMSO or 17-AAG. Furthermore, the results show that in the presence of 17-AGG, viral protein synthesis levels are decreased compared to DMSO-treated control; however, viral proteins are still detectable for the four RVFV proteins (L, Gn/Gc, N and NSs), suggesting that protein synthesis is still active.

Since HSP90 is a chaperone protein that normally aids in proper protein folding and stability, we assessed the stability of the viral proteins in the presence of 17-AAG. Our pulse-chase analysis shows that the levels of L protein significantly decrease by 2 and 4 hpi in cells treated with 17-AAG as compared to DMSO-treated control (**Figure 2B**). At 2 hpi, only 17 ± 11% of labeled L protein remains compared to 81 ± 20% in the DMSO-treated cells, and by 4 hpi 6 ± 15% remains compared to 43 ± 17% in DMSO-treated cells. By contrast, the levels of Gn/Gc, N, and NSs proteins did not show a significant difference between the DMSO-treated and 17-AAG-treated cells (**Figure 2B**). Together, these findings demonstrate that, as shown earlier, HSP90 is important for maintenance of viral proteins levels in infected cells. Most importantly, they show that it serves a critical function in the stability of the viral L polymerase.

### HSP90 protects L protein from degradation by the proteasome

**Figure 3:**
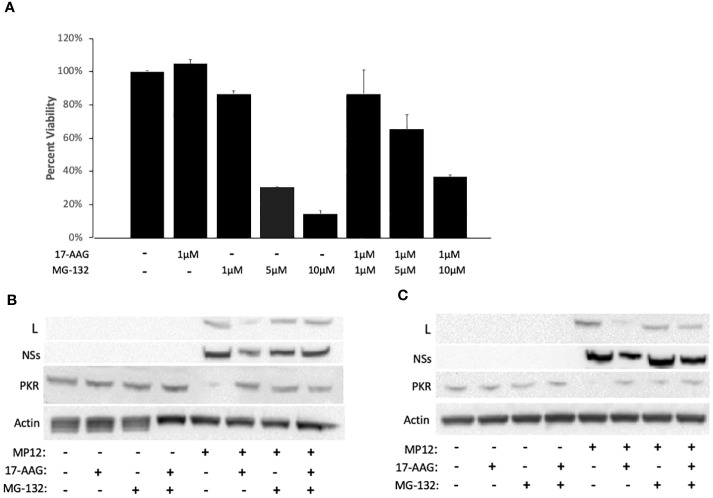
HSP90 Inhibition Leads to Degradation of the Viral L Polymerase through the Proteasome Pathway. **(A)** For cytotoxicity analysis, Vero cells were treated with either 17-AAG (1 μM), or MG-132 (1 μM, 5 μM, or 10 μM), or in combination (1 μM 17-AAG combined with 1 μM, or 5 μM, or 10 μM of MG-132). Mean values ±SEM from three biological replicates are shown. **(B, C)** Vero cells were treated both pre- and post-infection with MP12-L-V5, NSs-FLAG (MOI 3) using the following conditions: 17-AAG (1 μM) and MG-132 (1 μM); 17-AAG only (1 μM); MG-132 only (1 μM); DMSO (vehicle) control. Whole cell lysates were collected at 16 hpi **(B)** and 24 hpi **(C)** and the levels of PKR protein, L protein, and NSs protein were assessed by western blot. All signals were normalized to β-Actin.

Previous studies have demonstrated that disruption of the HSP90-client interaction can lead to degradation of client proteins by the cell proteasome (Loo et al., 1998; Isaacs et al., 2002; Shay et al., 2005). Therefore, we investigated whether instability of the viral L polymerase in the absence of HSP90 function involves degradation of L through the proteasome degradation pathway. To assess this, we used the well-established proteasome pathway inhibitor MG-132 to analyze its effects on viral protein stability (**Figure 3**). We first carried out MG-132 titration analysis to assess the cytotoxicity of MG-132 alone and also in conjunction with 17-AAG. The results demonstrate that at a concentration of 1 µM, MG-132 has no cytotoxic effects on Vero cells and also that co-treatment with 1 µM MG-132 together with 1 µM 17-AAG showed lack of cytotoxicity (**Figure 3A**). However, at higher MG-132 concentrations of 5 µM and 10 µM, the cell viability dropped to ~30% and ~10% respectively, and co-treatment of MG-132 and 17-AAG at higher concentrations also led to lower cell viabilities of about 65% and 40%, respectively (**Figure 3A**). Therefore, we used MG-132 at a final concentration of 1 µM for our studies.

To demonstrate that MG-132 treatment is causing proteasome inhibition, we also quantified PKR levels in infected cells as a control since it is known that the NSs protein of RVFV induces degradation of PKR through the proteasome pathway (Mudhasani et al., 2016). As expected, RVFV infection led to a decrease in PKR levels but the PRK degradation was reversed in infected cells pre-treated with MG-132, at both 16 hpi and 24 hpi (**Figures 3B, C**), demonstrating MG-132 inhibition of the proteasome pathway. Consistent with the findings of **Figures 1C** and **2B**, cells treated with 17-AAG showed decreased L polymerase levels compared to untreated infected cells; however, MG-132 treatment in addition to 17-AAG reversed this phenotype, giving rise to a noticeable increase in the levels of L protein at both 16 and 24 hpi (**Figures 3B, C**). These results point to the involvement of the proteasome pathway in degradation of the L protein and demonstrate that improved stability of the L polymerase by HSP90 is through inhibition of its degradation by the proteasome. They also suggest formation of a complex between HSP90 and the L polymerase to prevent L degradation through the proteasomal pathway.

### HSP90 protein co-localizes with the viral L polymerase and is part of a protein complex with L

**Figure 4:**
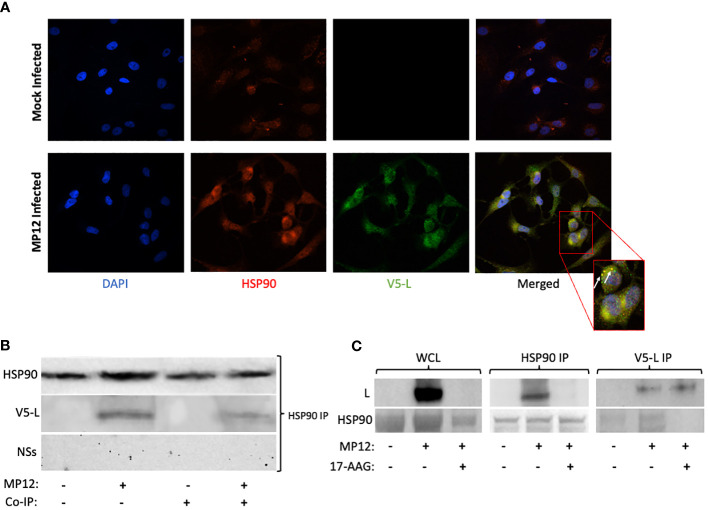
HSP90 Co-localizes with the Viral L Polymerase and Is Part of a Protein Complex with L. **(A)** Vero cells were infected with RVFV MP12-L-V5, NSs-FLAG (MOI 3) for 24 hours and were subsequently fixed and permeabilized for analysis by confocal immunofluorescence microscopy, using anti-V5 antibody for detection of L protein (Green) and anti-HSP90 antibody (red), with co-localization of the two signals resulting in yellow fluorescent signal. **(B, C)** Vero cells with or without treatment with 17-AAG were either left uninfected or were infected with MP12-L-V5, NSs-FLAG (MOI 3) for 24 hours, and whole cell lysates were analyzed by co-immunoprecipitation as described in the methods section. Briefly, target proteins were immunoprecipitated from whole cell lysates using magnetic Dynabeads coated with the specific antibody (anti-V5 or anti-HSP90). Following isolation, proteins were analyzed by western blot analysis using anti-V5, anti-NSs, or anti-HSP90 antibodies.

Given these findings, and because of the known interaction of HSP90 with the polymerase of some other non-retroviral ssRNA viruses, we next analyzed whether HSP90 forms a complex with the L protein to prevent its degradation through the proteasomal pathway. To assess this, we performed immunofluorescence studies of Vero cells infected with L-V5 tagged RVFV and immunolabeled for detection of V5-tagged L and HSP90 proteins. For this colocalization analysis, ten different field of cells, with each field containing at least 10 cells, were analyzed. The results show strong co-localization of HSP90 and the L protein in RVFV-infected cells (**Figure 4A**). Furthermore, to confirm the immunofluorescence results, co-immunoprecipitation (Co-IP) analysis was also performed. Using gentle lysis conditions designed to keep protein-protein interactions intact, whole cell lysates were prepared from uninfected cells as control and also from both infected Vero cells without any prior HSP90 inhibition and infected Vero cells in which HSP90 was inhibited by prior 17-AAG treatment. The lysates were then subjected to IP pull-down using either anti-HSP90 or anti-V5 antibodies. As anticipated, the pull-down using anti-HSP90 antibody resulted in successful immunoprecipitation of HSP90 from all cell lysates, and western blot probing with anti-V5 antibody demonstrated the presence of tagged L protein in the immunoprecipitated complex from infected cells without any prior HSP90 inhibition (**Figure 4B**). In contrast, the presence of the NSs protein was not detected (**Figure 4B**), suggesting that in agreement with our pulse-chase results, HSP90 interaction seems specific to the L protein of RVFV. The formation of a HSP90-L protein complex was also confirmed by reverse immunoprecipitation from infected cells without prior HSP90 inhibition. Thus, the pull-down using anti-V5 antibody, which resulted in successful immunoprecipitation of V5-L from the cell lysates, showed the presence of HSP90 in the immunoprecipitated complex (**Figure 4C**; V5-L IP).

To provide even further evidence of HSP90-L interaction as part of a complex, we also performed Co-IP pulldowns from infected cells in which HSP90 had been inhibited by pre-treatment with 17-AAG. Under these conditions, the presence of L protein was hardly detected in the Co-IP complex pulled down using anti-HSP90 antibody (**Figure 4C**; HSP90 IP condition, top row). This is consistent with both a significant lowering of the HSP90 protein in cells pre-treated with 17-AAG (**Figure 4C**; WCL, bottom row) and also a significant reduction in the levels of the L protein, as shown in **Figure 1C** and also by the whole cell lysate (WCL) western blot shown in **Figure 4C** (top row). In contrast, as expected, the reverse IP with anti-V5 antibody pulled down the L protein with or without prior 17-AAG inhibition (**Figure 4C**; V5-L IP, top row) whereas HSP90 was not detected under conditions of 17-AAG inhibition prior to infection (**Figure 4C**; V5-L IP, bottom row). Together with the immunofluorescence study findings, the Co-IP results demonstrate that HSP90 and RVFV L polymerase interact, either directly or indirectly, as part of a protein complex.

### Analysis of HSP90 inhibition effects using a virulent strain of RVFV

**Figure 5:**
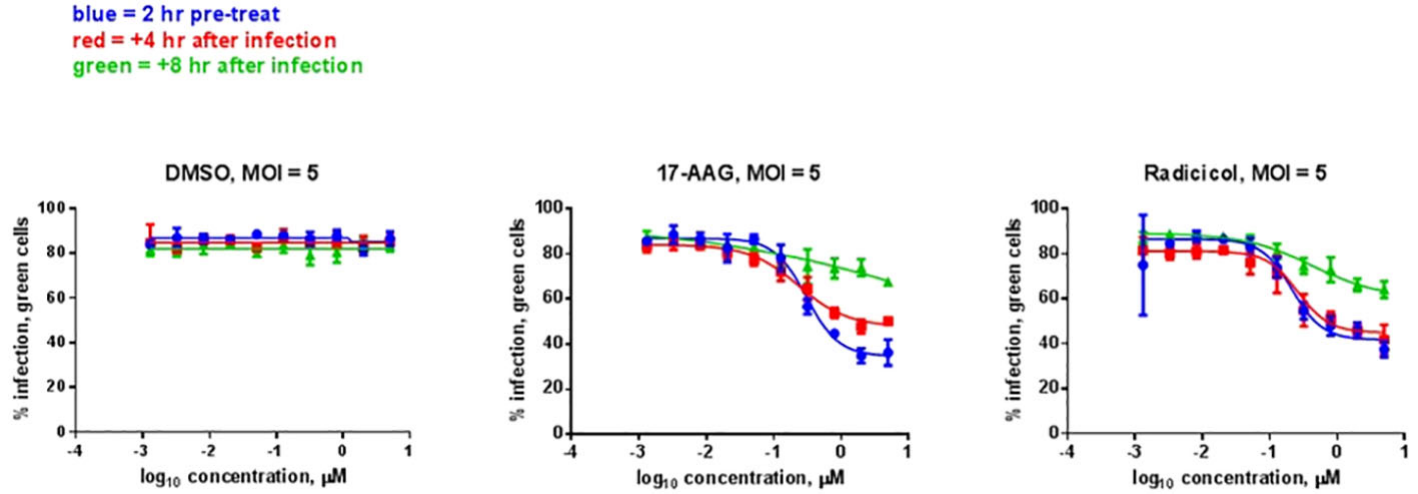
HSP Inhibitor Time of Addition Effects on ZH501. HepG2 cells were treated with HSP90 inhibitors 17-AAG or radicicol at the indicated times relative to infection and were left remaining in the culture media during the course of the experiment. DMSO was used as a control treatment. Cells were infected with RVFV ZH501 at MOI 5. At 20 hpi, measurement of RVFV infection was achieved by immunofluorescence microscopy and high content imaging and normalized to DMSO signal. Mean values ±SEM from three biological replicates are shown.

We analyzed whether the HSP90 effect on RVFV production is also observed with the virulent ZH501 strain of RVFV. Time of addition studies using 17-AAG, as well as the natural product radicicol which is another well-known HSP90 inhibitor, were performed to test their effects on replication of the ZH501 strain. Similar to 17-AAG, radicicol inhibits the ATPase activity of HSP90 through binding to the N-terminal ATP binding pocket to induce a conformational change. For these studies, HepG2 cells were treated with DMSO (control) or with 17-AAG or radicicol, either at two hours pre-infection, or at 4 hpi, or 8 hpi. Subsequently, the levels of viral N protein were analyzed at 20 hpi by immunofluorescence microscopy and high content imaging. For these experiments, we determined cytotoxicity in HepG2 cells after treatment with various concentrations of the two inhibitors. As discussed earlier, 17-AAG treatment at 1 μM concentration does not cause any cytotoxicity in HepG2 cells (**Figure 1A**). We also determined that the same applies for treatment of HepG2 cells with radicicol, as treatment of HepG2 cells with increasing radicicol concentrations up to 1 μM does not cause any cytotoxicity and cytotoxic effects are observed only at the higher 10 μM concentration (**Supplementary Figure 1**). Based on these observations, percent infection of cells treated with increasing concentrations of the inhibitors from 1 nM to 1 µM were determined both pre-and post-infection (**Figure 5**). DMSO treatment (left panel) both pre- and post-infection had no effect on the degree of infection, giving an approximately 80% infection rate of HepG2 cells at all DMSO concentrations analyzed. On the other hand, 17-AAG treatment (middle panel) resulted in clear viral inhibitory effects at 100 nM concentrations and higher, with the most significant change occurring when the inhibitor was given 2 hours pre-infection (about 40% infection rate). When 17-AAG was given at 4 or 8 hpi, the infection rate rose to about 50% and 70%, respectively, at 1 µM concentration. Similar results were observed with radicicol treatment; treatment at 2 hours pre-infection and 4 hpi showed similar infection rates of about 40% at 1 μM, whereas treatment at 8 hpi gave a 70% infection rate. Taken together, these results demonstrate the importance of HSP90 function for viral replication during infection with virulent RVFV, and show that RVFV inhibition is most effective when HSP90 function is inhibited either pre-infection or soon after infection of cells.

## Discussion

There exists a continuing need for urgent development of effective therapies and vaccines for emerging infectious diseases such as RVF. Traditionally, the development of therapeutics against viral agents has mainly focused on targeting viral factors. However, in more recent efforts, there has been an increase in approaches that rely on targeting host factors hijacked by the virus. Since virions do not possess the necessary machinery to replicate on their own, they must rely on host proteins to carry out most of their functions, and several host factors have been identified that are involved in the progression of a variety of viral infections (Austin et al., 2012). In particular, HSP90 has been shown to play a role in the viral life cycle of a number of viruses such as the Influenza virus, Herpes simplex virus, Flock house virus, Paramyxovirus, and the Mumps virus (Burch and Weller, 2005; Kampmueller and Miller, 2005; Connor et al., 2007; Jirakanwisal et al., 2015; Katoh et al., 2017).

In our previous study, in-depth proteomic characterization of host proteins associated with RVFV virions led to identification of several heat shock chaperones, including HSP90, as virion-associated host factors that are important for virus production. At basal levels, HSP90 protects against protein misfolding and aggregation, as well as other proteotoxic stressors such as heavy metals, hypoxia, acidosis, and invading pathogens. Consistent with these pleiotropic influences, HSP90 has also been implicated in various types of cancers (Cohen and Esfahani, 2016; Wu et al., 2017; Wang et al., 2017; Wang Y. et al., 2017), and HSP90 inhibition has been explored as a therapeutic option for cancer treatment. Consequently, a handful of HSP90 inhibitors have advanced to phase 2 or phase 3 clinical trials and have been well characterized. For these reasons, HSP90 serves as an attractive target among the host proteins of interest that were identified in our proteomic analysis.

In our previous study, we demonstrated that inhibition of HSP90 significantly reduces RVFV titers without compromising the functional capacity of released virions. In the current study, we have extended our previous findings by investigation of the mechanisms by which HSP90 regulates RVFV production. We have found that inhibition of HSP90 in infected cells causes a significant decrease in both viral RNA as well as viral protein levels starting at the very early stages of infection, suggesting the involvement of HSP90 in viral genome replication and/or transcription.

Given the dramatic decrease in viral protein levels under conditions of HSP90 inhibition and the known role of HSP90 in preventing client protein degradation, we performed pulse-chase studies to analyze HSP90 effects on viral protein stability. Prevention of protein degradation by host chaperone, and specifically HSP90, has been demonstrated in other virus infections (Connor et al., 2007; Smith et al., 2010; Munday et al., 2015). In Vesicular Stomatitis Virus, for example, inhibition of HSP90 protected the L polymerase from degradation (Isaacs et al., 2002). This is similar to our findings with RVFV. While inhibition of HSP90 had very little effect on the stability of N, NSs, and Gn/Gc proteins, it nevertheless significantly affected the stability of RVFV L polymerase (**Figure 2**). Mechanistically, this further demonstrates that HSP90 serves as a critical host factor for viral genome transcription and replication since they require the activity of the L polymerase. This has also been shown previously for a wide spectrum of negative-stranded RNA viruses and some positive-strand RNA viruses (Momose et al., 2002; Kampmueller and Miller, 2005; Connor et al., 2007; Braga et al., 2017; Liu et al., 2017). Our results also show that the HSP90-dependent stability of L is through prevention of degradation by the proteasome pathway. As shown in **Figure 3**, inhibition of the proteasome by MG-132 showed decreased L polymerase degradation in cells treated with both 17-AAG and MG-132, as compared to cells that were treated with 17-AAG alone. This finding is also consistent with other reports in which inhibition of HSP90 leads to degradation of HSP90 client proteins through the proteasomal pathway (Bagatell et al., 2001; Fan et al., 2005; Shay et al., 2005; Wang Z. et al., 2017). In this regard, our results have also identified the L polymerase of RVFV as a client protein of HSP90 through Co-IP and colocalization imaging studies (**Figure 4**).

While we have shown that the HSP90 effects on viral production are also observed with the virulent ZH501 strain (**Figure 5**), consistent with many previous RVFV publications the mechanistic studies were performed using the attenuated MP12 strain of RVFV. The MP12 strain is derived from the wild-type pathogenic strain ZH548 and contains various mutations across all three genome segments (S, M, and L). Although we have shown the importance of HSP90 activity for both pathogenic RVFV and the attenuated MP12 strain, future mechanistic studies using virulent strains of RVFV such as ZH501 are warranted to compare with, and expand, the mechanistic details that we have shown here using MP12. For instance, it will be of interest to map out the protein interaction regions between L and HSP90 and analyze whether any of the MP12 mutations in the L segment influence the type or strength of this interaction. Furthermore, as our previous proteomic analysis revealed that other HSP proteins may also be involved during RVFV infection, future studies are needed to determine their potential contributions and whether they may be involved differently when comparing MP12 and virulent strains.

**Figure 6:**
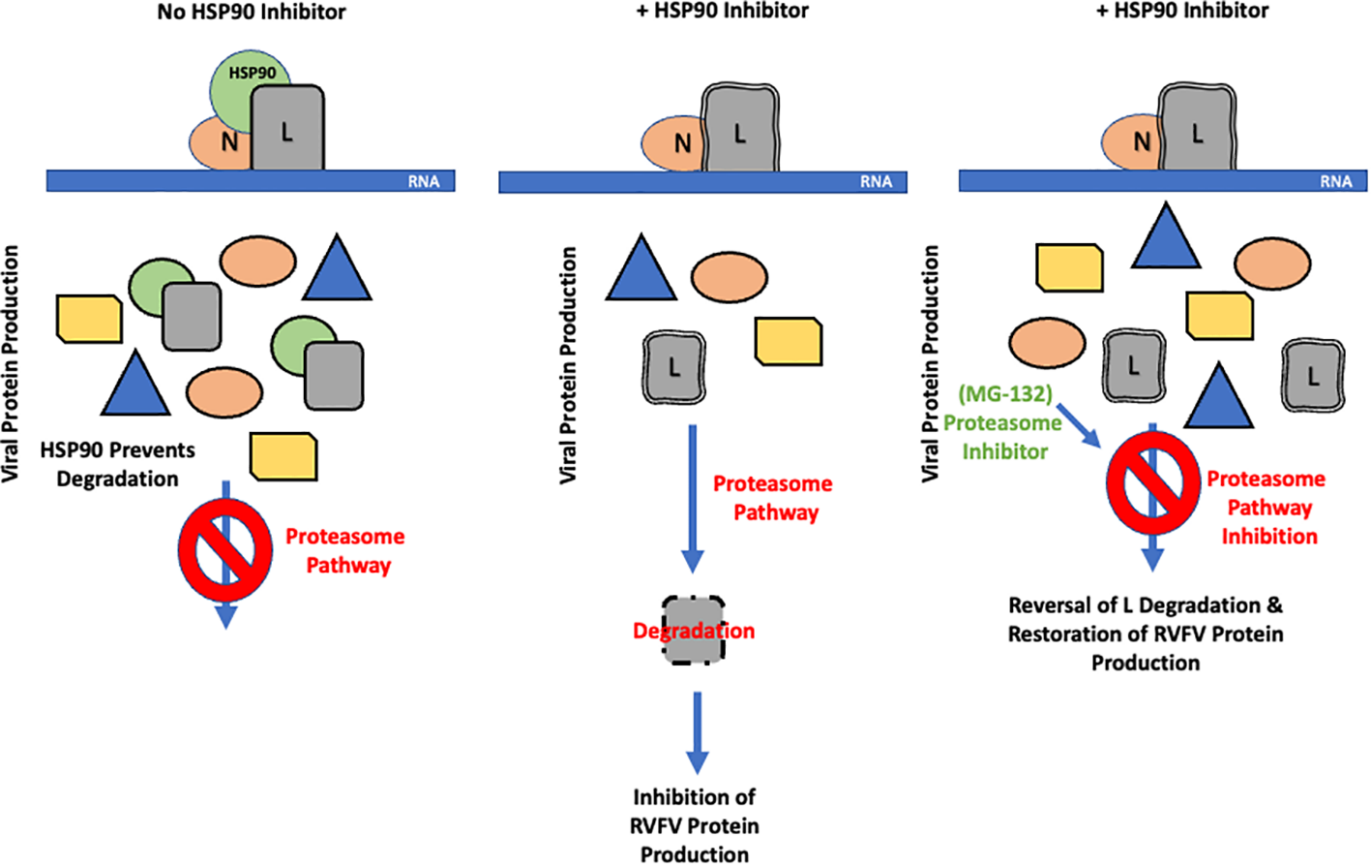
Proposed Model of Viral RNA Polymerase Protection from Degradation by the Host HSP90 Protein. Following RVFV entry into the host cell, HSP90 interacts with the L protein as part of a protein complex and accompanying conformational changes in HSP90 that are coupled to ATP binding and hydrolysis confers increased stability to L, thus allowing increased viral genome transcription and production of viral proteins and increased viral replication. Considering the very large size of the L protein and based on the results of the heat shock experiment presented here, this conferred stability may be a result of HSP90 functioning as a folding chaperone for the L polymerase. In the absence of HSP90 function, its ability to stabilize L is compromised, resulting in decreased L stability and degradation of L through the proteasome, an effect that can be reversed through inhibition of proteasome activity.

Based on our findings, we propose the model shown in **Figure 6**. In this model, soon after viral entry, HSP90 forms a complex with the L polymerase of RVFV, thereby stabilizing it and preventing its degradation through the proteasome pathway. Considering that L protein is very large (~220kDa) and would require chaperone assistance for proper folding, and as suggested by our heat shock assay results, it is possible that HSP90 confers stability to L by acting as a folding chaperone, an aspect that needs to be investigated in future mechanistic studies, This protection of L from degradation allows for increased viral genome transcription and replication that ultimately leads to increased production of the full complement of the viral proteins and thereby an increased viral load. These studies highlight the potential of HSP90 inhibitors for development of therapeutics against RVFV infection and warrant *in vivo* efficacy studies of these inhibitors against RVFV infection in future studies. Such potentially effective host-based approaches could complement recent promising advances, such as demonstration of potent neutralizing activity of a human antibody that prevents vertical RVFV transmission in a rat model (McMillen et al., 2023). In this regard, 17-AAG is a well-studied HSP90 inhibitor that is well tolerated at biologically active doses and has progressed to phase III clinical trials (Magwenyane et al., 2022). Accordingly, 17-AAG may serve as a suitable therapeutic candidate for RVFV infections, allowing virus inhibition at the early stages of the virus life cycle.

## Data availability statement

The original contributions presented in the study are included in the article/**Supplementary Material**. Further inquiries can be directed to the corresponding author.

## Ethics statement

Ethical approval was not required for the studies on humans in accordance with the local legislation and institutional requirements because only commercially available established cell lines were used. Ethical approval was not required for the studies on animals in accordance with the local legislation and institutional requirements because only commercially available established cell lines were used.

## Author contributions

FA: Writing – review & editing, Writing – original draft, Visualization, Validation, Methodology, Investigation, Formal analysis. AB: Writing – original draft, Validation, Methodology, Investigation. KT: Writing – review & editing, Validation, Methodology, Investigation, Formal analysis, Conceptualization. SO: Writing – review & editing, Validation, Investigation. KK-H: Writing – review & editing, Supervision, Methodology. CS: Writing – review & editing, Supervision, Resources, Methodology, Conceptualization. SB: Writing – review & editing, Supervision, Resources. SM: Writing – review & editing, Supervision, Resources, Methodology, Funding acquisition, Conceptualization. RH: Writing – review & editing, Visualization, Supervision, Resources, Project administration, Methodology, Funding acquisition, Conceptualization.

## Supplementary material

Supplementary Figure 1 Analysis of Radicicol Toxicity for HepG2 Cells. HepG2 cells were treated with either radicicol at various concentrations (100 pM, 1 nM, 10 nM, 100 nM, 1µM, or 10 µM), or an equivalent volume of DMSO vehicle (Negative Control condition), or a toxic volume of DMSO (Positive Control condition). Cell viability was measured at 24 hours post treatment (hpt) by the CellTiter-Glo® Luminescent Cell Viability Assay; ***P = 0.0001; ****P<0.0001.
