# Supplementary figures and images for: HSP90 is part of a protein complex with the L polymerase of Rift Valley fever phlebovirus and prevents its degradation by the proteasome during the viral genome replication/transcription stage
Source: Front Cell Infect Microbiol. 2024 May 10;14:1331755. doi: 10.3389/fcimb.2024.1331755 (PMC11127626; doi:10.3389/fcimb.2024.1331755)

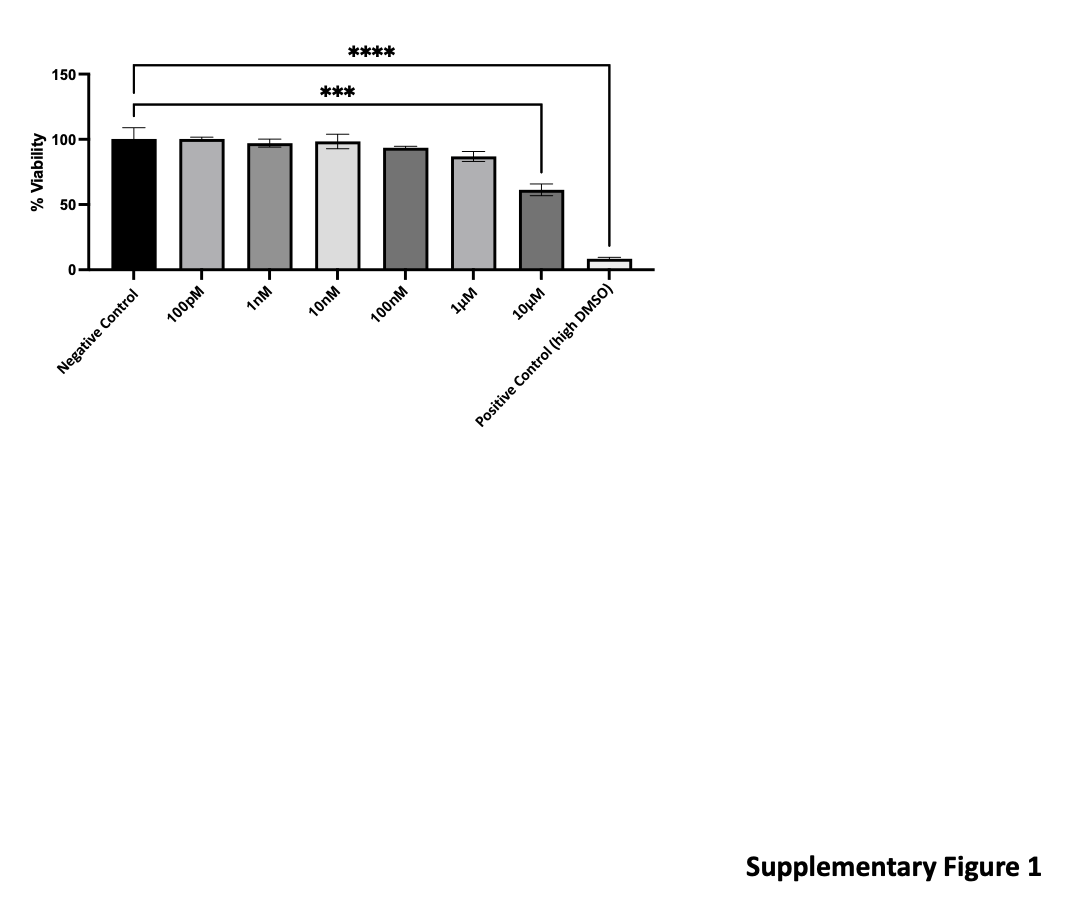

Supplement: Supplementary Figure 1 — Analysis of Radicicol Toxicity for HepG2 Cells. HepG2 cells were treated with either radicicol at various concentrations (100 pM, 1 nM, 10 nM, 100 nM, 1µM, or 10 µM), or an equivalent volume of DMSO vehicle (Negative Control condition), or a toxic volume of DMSO (Positive Control condition). Cell viability was measured at 24 hours post treatment (hpt) by the CellTiter-Glo® Luminescent Cell Viability Assay; ***P = 0.0001; ****P<0.0001. [file Image_1.tiff]
